# The inhibition of de novo purine synthesis increases LAMP2 expression to preserve cell viability

**DOI:** 10.1038/s41420-025-02884-0

**Published:** 2025-12-11

**Authors:** Angela De Cristofaro, Serena Castelli, Federica Felice, Maria Rosa Ciriolo, Enrico Desideri

**Affiliations:** 1 Department of Biology, University of Rome “Tor Vergata”, Via Della Ricerca Scientifica, Rome, Italy; 2 Department of Human Sciences and Promotion of the Quality of Life, San Raffaele Roma Open University, Via di Val Cannuta, 247, Rome, Italy; 3 IRCCS San Raffaele Roma, Via di Val Cannuta, 247, Rome, Italy; 4 Department of Life Sciences, Health and Health Professions, Link Campus University, Via del Casale di San Pio V, 44, Rome, Italy

**Keywords:** Lysosomes, Stress signalling

## Abstract

Cancer cells rewire their metabolism to sustain the high proliferative rate. Metabolism is therefore a common vulnerability of cancer cells, successfully exploited for therapeutic purposes. Intrinsic tumor characteristics and adaptive responses of cancer cells can however reduce the short and long-term efficacy of such a strategy. Understanding the determinants of therapy response and the mechanisms of chemoresistance is crucial to maximize therapy efficacy. In cancer, lysosomes undergo massive changes in their localization, size, and composition that support tumor progression. Additionally, lysosomes are one of the crucial drivers of chemoresistance via the drug sequestration or by facilitating adaptations to stress conditions. In the last decades, several reports have shown that lysosomal membrane proteins, such as the lysosome-associated membrane proteins 1 and 2 (LAMP1 and LAMP2), are deregulated in different cancer types and their expression has been correlated to drug efficacy. We performed an in silico gene essentiality and drug sensitivity screenings, revealing that LAMP2 expression is one of the determinants of resistance to inhibitors of de novo purine synthesis. In vitro experiments confirmed the in silico data and also showed that purine synthesis inhibitors trigger a ROS- and transcriptional-dependent increase of LAMP2. Our results identify the upregulation of LAMP2 expression as an adaptive response to purine synthesis inhibition to preserve cell viability and, in those tumors showing high LAMP2 levels, could also be an indicator of intrinsic resistance to these drugs that may be taken into consideration during the selection of the most appropriate therapy.

## Introduction

Lysosomes are acidic organelles whose primary function is the degradation of intracellular and extracellular material that represents an additional source of energy and building blocks for the cells [1]. In cancer, lysosomes undergo massive changes in their localization, size, and composition that support tumor progression [2, 3]. Additionally, lysosomes are one of the crucial drivers of chemoresistance via sequestration of drugs (drug safe house effect) or by facilitating adaptations to stress condition [4, 5]. In the last decades, several reports have shown that lysosomal membrane proteins, particularly lysosome-associated membrane proteins (LAMPs) 1 and 2 (LAMP1 and LAMP2), are deregulated in different cancer types [6, 7] and their expression has been associated with drug resistance [8, 9]. These proteins may therefore be able to act as predictive markers of drug efficacy.

Many cancer cells display a rearrangement of their metabolism to fulfill the increased need for energy and building blocks for the synthesis of macromolecules [10]. The high proliferative rate massively increases the demand for nucleotides, which are incorporated into DNA and ribosomal RNA (rRNA) and are required during DNA replication and ribosome biogenesis [11]. Cells can synthesize nucleotides via two pathways: the energy-efficient salvage pathway that uses intermediates of nucleotide degradation such as free bases to resynthesize nucleotide, or the de novo pathway, which produce new nucleotides from amino acids, carbon dioxide, folate derivatives, and which require great amounts of ATP [11]. While normal cells mostly rely on the salvage pathway for their needs, cancer cells upregulate de novo nucleotide synthesis to obtain sufficient nucleotide pools. Consistently, de novo nucleotide synthesis is found to be upregulated in several types of cancer, including hepatocellular carcinoma (HCC), one of the deadliest cancers worldwide [12]. Nucleotide biosynthesis is therefore a common metabolic vulnerability of cancer cells and is successfully used as a therapeutic target in some cancer types [13]. For instance, the antimetabolite 6-mercaptopurine (6-MP) has been granted approval by the FDA for the treatment of acute lymphoblastic leukemia. 6-MP inhibits phosphoribosyl pyrophosphate amidotransferase, the first step in de novo purine synthesis [14]. Other inhibitors are currently in clinical or pre-clinical testing [15]. Intrinsic tumor characteristics and adaptations to drug exposure can reduce the short and long-term efficacy of drugs and give rise to chemoresistance. Understanding the determinants of chemotherapeutic efficacy and the mechanisms responsible for chemoresistance is crucial for the selection of the most appropriate approach and for the improvement of therapy efficacy.

In this paper, we demonstrate that the inhibition of de novo purine synthesis specifically increases LAMP2 expression in HCC cells in a transcriptional- and ROS-dependent manner. Additionally, we also identify LAMP2 as a pro-survival factor upon drug exposure, whose modulation influences the toxic effect of purine synthesis inhibitors.

## Results

### In silico analyses identify LAMP2 expression as a predictive marker of resistance of cancer cells to the inhibition of the de novo purine synthesis

**Fig. 1:**
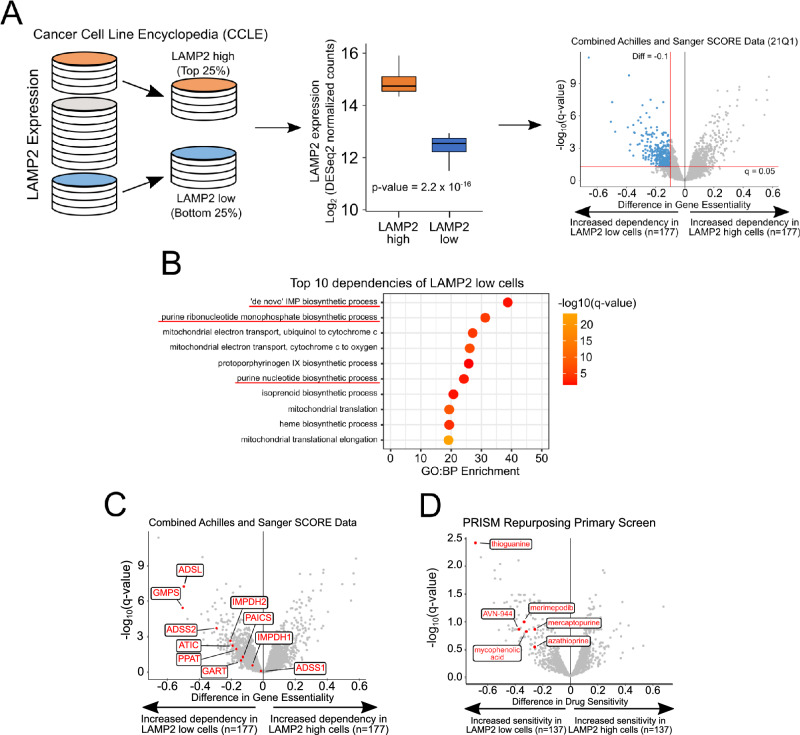
Identification of LAMP2-dependent cell vulnerabilities. **A** Schematic representation of the workflow used to identify genetic vulnerabilities dependent on LAMP2 expression. **B** Gene Ontology-Biological Processes (GO:BP) terms over-represented in LAMP2 low cells. Volcano plots of differential gene essentiality (**C**) and drug sensitivity (**D**) of LAMP2 low vs LAMP2 high cells. Red dots and labels represent genes involved in the de novo purine synthesis (**C**) or drugs targeting purine synthesis (**D**).

Lysosomal membrane proteins contribute to the chemoresistance of cancer cells [5]. To investigate whether the expression of LAMPs can be employed as a predictor marker of resistance to specific classes of drugs, we took advantage of the project Achilles CRISPR knockout screen, which contains genome-wide essentiality data for hundreds of cell lines [16]. Initially, we ranked the cell lines present in the CRISPR KO screening according to their LAMP2 expression, using gene expression data included in the Cancer Cell Line Encyclopedia (CCLE). We then identified two groups: LAMP2 low, which includes cells with the lowest LAMP2 expression (bottom 25%); and LAMP2 high, which includes cells with the highest LAMP2 expression (top 25%) (Fig. 1A; left panel). The two groups exhibit a difference in LAMP2 expression of nearly threefold (Fig. 1A; center panel). We then calculated the difference (LAMP2 low—LAMP2 high) of the mean gene essentiality between the two groups (Fig. 1A; right panel). To identify pathways whose essentiality inversely correlate with LAMP2 expression, we selected all genes with a mean differential essentiality ≤ –0.1 and an adjusted *p-*value (q-value) ≤0.05 and performed gene-enrichment analysis. The result indicates that GO Terms related to the de novo purine synthesis rank among the most enriched terms in the Biological Processes category (GO:BP) (Fig. 1B). Consistently, the analysis of the mean differential essentiality of the individual genes involved in the de novo purine synthesis shows that they are all more essential in LAMP2 low cells than in LAMP2 high cells (Fig. 1C). To corroborate our findings, we performed the same analysis using data included in the PRISM Repurposing Primary screening, which contains the results of viability screens of more than 4000 chemical compounds (https://depmap.org/repurposing/). Similarly to gene essentiality, cells expressing high LAMP2 levels are predicted to be less sensitive to all inhibitors of purine synthesis included in the PRISM Repurposing Primary screening (Fig. 1D). Notably, 6-thioguanine (6-TG) is the drug with the highest differential sensitivity between LAMP2 low and LAMP2 high cells. Our data suggest that LAMP2 expression can mediate cellular resistance to the inhibition of the de novo purine synthesis.

### LAMP2 expression influences the cytotoxic effect of inhibitors of the de novo purine synthesis

**Fig. 2:**
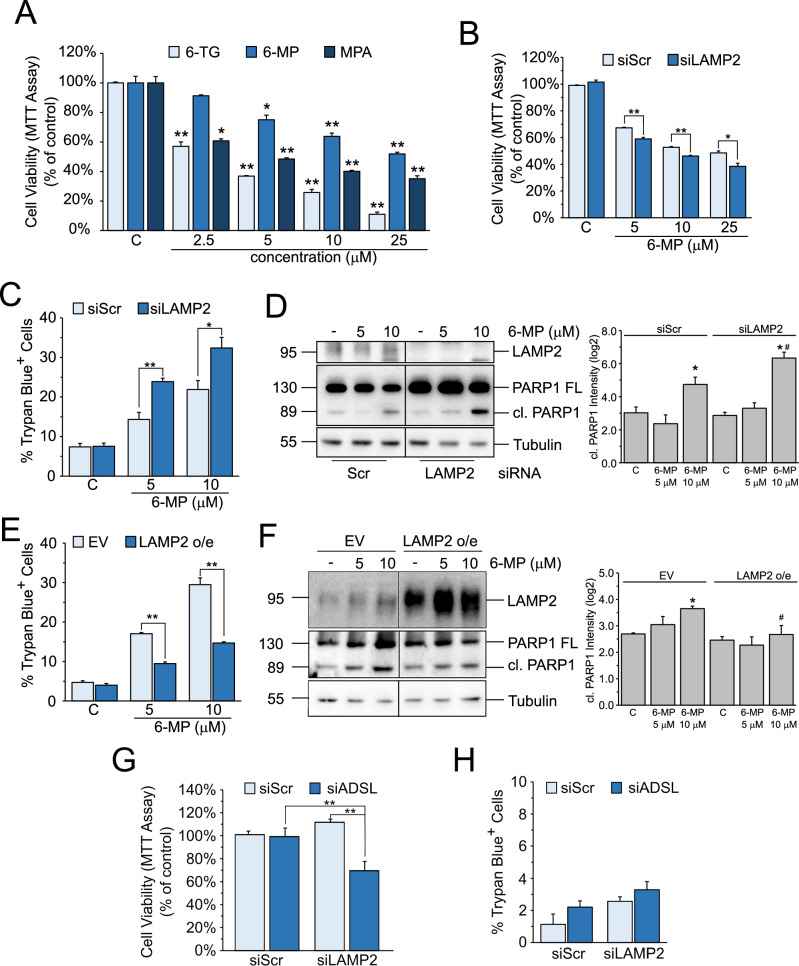
LAMP2 expression promotes resistance to inhibition of purine synthesis. **A** MTT assay in Hep3B cells treated for 48 h with the indicated drug concentrations. Data are expressed as the mean ± SEM of three independent experiments. *p ≤ 0.05; **p ≤ 0.01 vs C. **B** MTT assay in siScr or siLAMP2 Hep3B cells treated for 48 h with the indicated drug concentrations. Data are expressed as the mean ± SEM of three independent experiments. *p ≤ 0.05; **p ≤ 0.01 vs siScr. Trypan blue exclusion assay in LAMP2 knockdown (siLAMP2) (**C**) or LAMP2 overexpressing (LAMP2 o/e) (**E**) Hep3B cells treated for 48 h with the indicated drug concentrations. Data are expressed as the mean ± SEM of three independent experiments. *p ≤ 0.05; **p ≤ 0.01 vs siScr or EV. (left) Western blot analysis of PARP1 in LAMP2 knockdown (siLAMP2) (**D**) or LAMP2 overexpressing (LAMP2 o/e) (**F**) Hep3B cells treated for 48 h with the indicated drug concentrations. (right) Densitometric analysis of cleaved PARP1 band intensities. Data are expressed as the mean ± SEM of log2(normalized band intensity) of three independent experiments. *p ≤ 0.05 vs C; #*p* ≤ 0.05 vs siScr or EV. 6-MP: 6-mercaptopurine; 6-TG: 6-thioguyanine; MPA: mycophenolic acid. MTT (**G**) and Trypan blue exclusion (**H**) assays of Hep3B cells silenced for LAMP2, ADSL or both. Data are expressed as the mean ± SEM of three independent experiments. **p ≤ 0.01.

To validate the in silico data, we investigated in vitro whether LAMP2 modulation influences the sensitivity of cancer cells to inhibitors of the de novo purine synthesis. As the main cellular model, we selected the HCC cell line Hep3B, because purine synthesis was shown to be upregulated in HCC and it is a potential therapeutic target [17]. First, we treated Hep3B cells for 48 h with increasing concentrations of three purine synthesis inhibitors, 6-thioguanine (6-TG), 6-mercaptopurine (6-MP) or mycophenolic acid (MPA), and performed an MTT cell viability assay. All inhibitors significantly reduced cell viability at low micromolar doses, with 6-TG being the most toxic of the three (Fig. 2A). For the following experiments we mainly used 6-MP, the one showing the lowest toxicity. Next, we silenced LAMP2 with a specific siRNA and treated siScr and siLAMP2 Hep3B cells with 6-MP for 48 h. MTT assay shows that LAMP2 silencing significantly reduces cell viability (Fig. 2B) and it is paralleled by an increase in cell death, evidenced by the increased number of Trypan blue positive cells (Fig. 2C) and the more robust expression of the cleaved form of PARP-1, a caspase 3 substrate (Fig. 2D). On the contrary, LAMP2 overexpression reduces Trypan blue positive cells (Fig. 2E) and cleaved PARP-1 (Fig. 2F) upon 6-MP treatment.

Finally, to further confirm the role of LAMP2 in the resistance to purine synthesis inhibition we knockdown ADSL, one of the genes involved in de novo purine synthesis, in siScr and siLAMP2 cells. Cell viability analysis shows that only the double LAMP2 and ADSL knockdown is able to significantly reduce cell viability while the single knockdown of either siLAMP2 or siADSL does not have any effect (Fig. 2G). Unlike 6-MP, siADSL does not induce cell death, neither alone nor in combination with siLAMP2 (Fig. 2H), indicating that the reduced viability is due to a cytostatic effect.

These results confirm the bioinformatics data and indicate that LAMP2 expression contributes to cellular resistance to inhibition of purine synthesis.

### LAMP2-dependent resistance to inhibition of purine synthesis is independent of autophagy

**Fig. 3:**
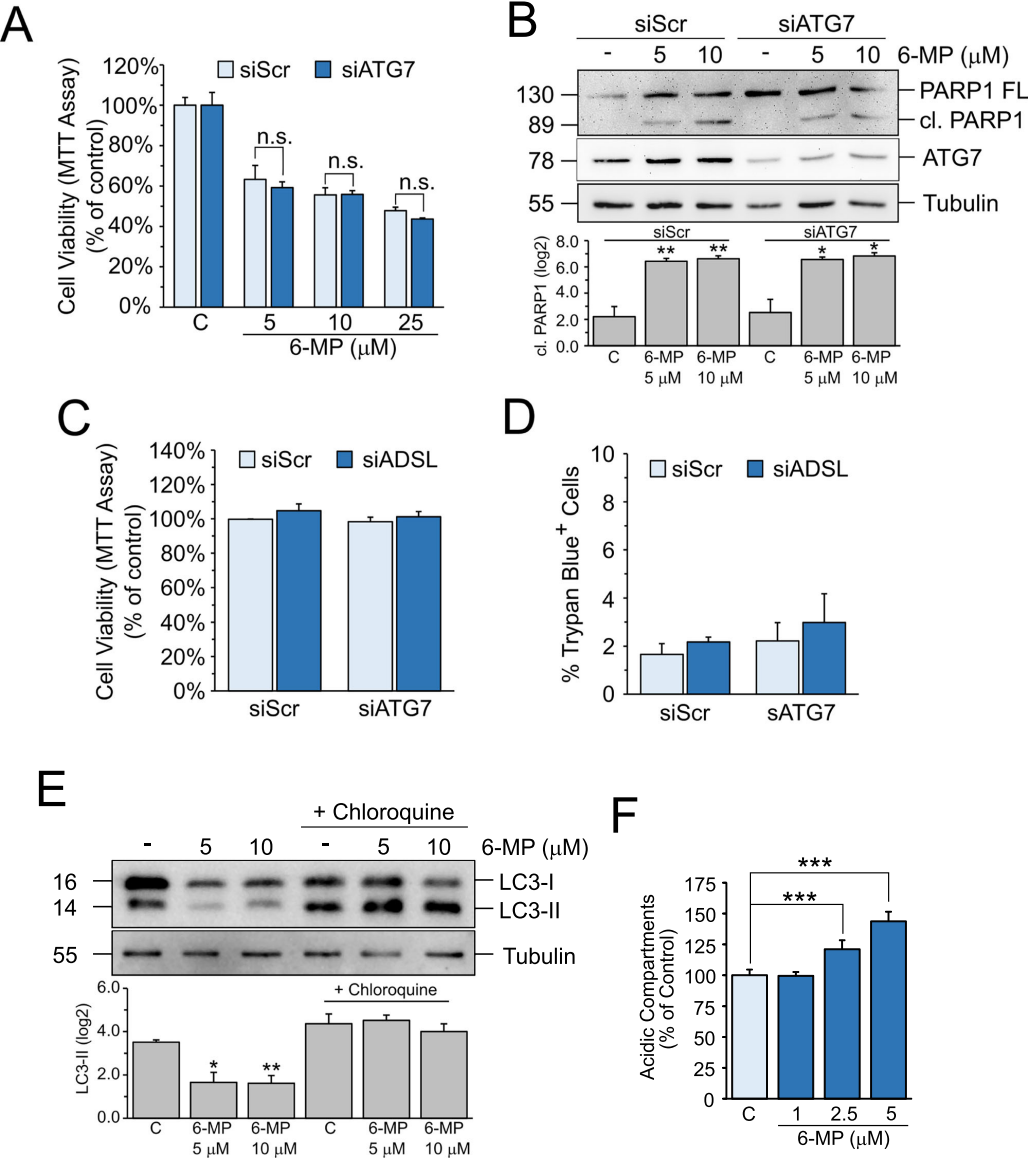
LAMP2-mediated resistance to inhibition of purine synthesis is independent of autophagy. **A** MTT assay in siScr or siATG7 Hep3B cells treated for 48 h with the indicated drug concentrations. Data are expressed as the mean ± SEM of three independent experiments. *p ≤ 0.05; **p ≤ 0.01 vs siScr. **B** (upper) Western blot analysis of PARP1 in siScr and siATG7 Hep3B cells treated for 48 h with the indicated drug concentrations. (lower) Densitometric analysis of cleaved PARP1 band intensities. Data are expressed as the mean ± SEM of log2(normalized band intensity) of three independent experiments. *p ≤ 0.05; **p ≤ 0.05 vs C. MTT (**C**) and Trypan blue exclusion (**D**) assays of Hep3B cells silenced for LAMP2, ADSL or both. Data are expressed as the mean ± SEM of three independent experiments. **E** (upper) Western blot analysis of LC3 in Hep3B cells treated for 48 h with the indicated drug concentrations in the presence or absence of 50 μM chloroquine for the last 4 h. (lower) Densitometric analysis of cleaved PARP1 band intensities. Data are expressed as the mean ± SEM of log2(normalized band intensity) of three independent experiments. *p ≤ 0.05; **p ≤ 0.01 vs C. **F** Cytofluorometric analysis of intracellular acidification using Acridine Orange in Hep3B cells treated for 48 h with the indicated drug concentrations. Data are expressed as the mean ± SEM of three independent experiments. ***p ≤ 0.001 vs C. 6-MP: 6-mercaptopurine.

At the lysosomes, LAMP2 plays several functions, including the regulation of autophagy [18]. To test whether autophagy plays a role in the resistance to inhibition of purine synthesis, we performed siRNA-mediated knockdown of ATG7, a protein involved in the initial phases of autophagosome formation, and treated Hep3B cells with 6-MP. MTT cell viability assay and WB analysis of cleaved PARP-1 indicate that, unlike LAMP2, the inhibition of autophagy does not influence the cytotoxicity of 6-MP (Fig. 3A, B). Similarly, ATG7 knockdown was unable to reduce cell viability of siADSL cells (Fig. 3C, D), differently from what we observed in siLAMP2 cells (Fig. 2G, H).

The analysis of autophagic flux upon 6-MP treatment shows that 6-MP strongly decreases both LC3-I and LC3-II, the latter being the lipidated form of LC3 and a commonly used marker of autophagosomes (Fig. 3E). LC3-II levels are, however, comparable in 6-MP-treated and control cells when the late-stage autophagy inhibitor chloroquine was used. These results indicate that autophagy is not induced but there is an increased degradation rate of autophagosomes, possibly due to enhanced lysosomal activity. FACS analysis shows a dose-dependent increase in intracellular acidification, which mostly depends on lysosomes, in Hep3B cells treated with 6-MP and stained with Acridine Orange (Fig. 3F), in agreement with the hypothesis of an increased lysosomal activity. Altogether, these data indicate that cells rely on the lysosomes and not on autophagy to counteract the toxic effects of 6-MP.

### Inhibition of de novo purine synthesis increases LAMP2 expression

**Fig. 4:**
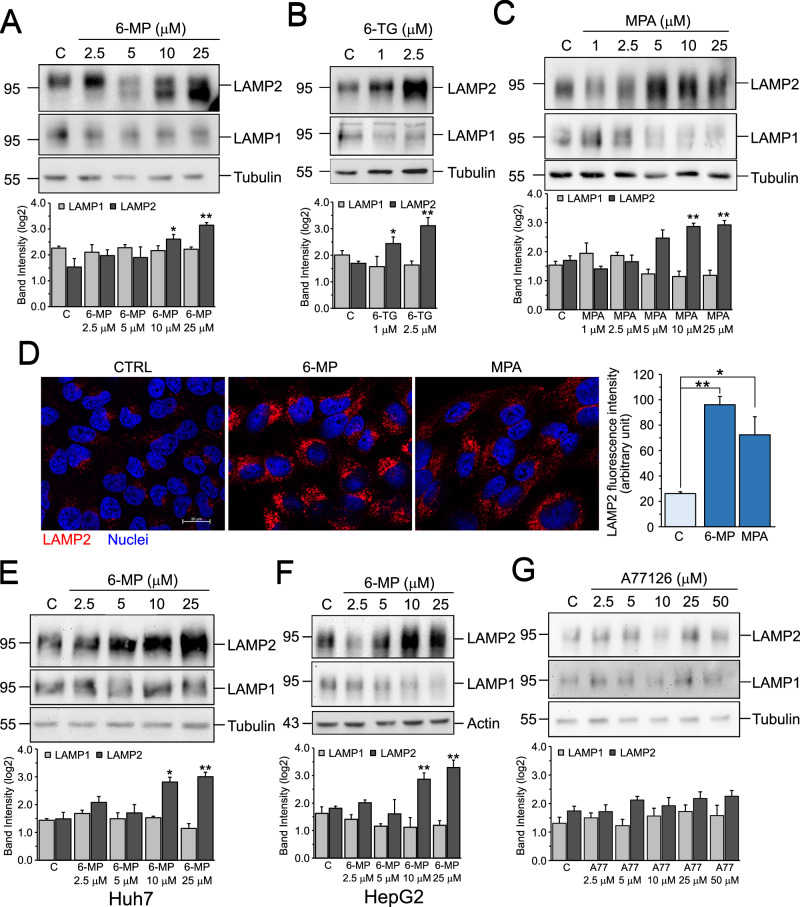
Inhibition of purine synthesis increases LAMP2 expression. **A–C** (upper) Western blot analysis of LAMP1 and LAMP2 in Hep3B cells treated for 48 h with the indicated drug concentrations. (lower) Densitometric analysis of cleaved PARP1 band intensities. Data are expressed as the mean ± SEM of log2(normalized band intensity) of three independent experiments. *p ≤ 0.05; **p ≤ 0.01 vs C. **D** Immunofluorescence analysis of LAMP2 in Hep3B cells treated for 48 h with 10 μM of either 6-MP or MPA. Data are expressed as the mean ± SEM of fluorescence intensity of 50 cells from three independent experiments. *p ≤ 0.05 vs C; **p ≤ 0.01 vs C. (Upper) Western blot analysis of LAMP1 and LAMP2 in HuH7 (**E**) and HepG2 (**F**) cells treated for 48 h with the indicated drug concentrations. (lower) Densitometric analysis of LAMP1 and LAMP2 band intensities. Data are expressed as the mean ± SEM of log2(normalized band intensity) of three independent experiments. *p ≤ 0.05 vs C; **p ≤ 0.01 vs C. **G** (Upper) Western blot analysis of LAMP1 and LAMP2 in Hep3B cells treated for 48 h with the indicated concentrations of A77126. (lower) Densitometric analysis of LAMP1 and LAMP2 band intensities. Data are expressed as the mean ± SEM of log2(normalized band intensity) of three independent experiments. 6-MP 6-mercaptopurine, 6-TG 6-thioguanine, MPA mycophenolic acid.

We have so far shown that LAMP2 expression influences the cytotoxic effect of 6-MP in an autophagy-independent manner. The increased intracellular acidification and the accelerated degradation rate of autophagosomes upon 6-MP treatment suggest a direct effect of purine synthesis inhibition on lysosomes. First, we treated Hep3B cells for 48 h with increasing concentration of 6-MP and analyzed the expression of LAMP1 and LAMP2, the two most abundant lysosomal membrane proteins. Figure 4A shows that 6-MP increases the expression of LAMP2, but not of LAMP1, in a dose-dependent manner. Similar results have been obtained with two additional inhibitors, 6-TG and MPA (Fig. 4B, C), indicating that inhibition of purine synthesis specifically acts on LAMP2. Immunofluorescence analysis of LAMP2 in cells treated for 48 h with either 6-MP or MPA shows a significant increase in LAMP2 fluorescence intensity (Fig. 4D), confirming that LAMP2 is increased under these conditions. We then treated two additional cell lines, Huh7 and HepG2, with 6-MP for 48 h to test whether the accumulation of LAMP2 also occurs in other cells. Similarly to Hep3B, 6-MP induces a dose-dependent increase of LAMP2 in both cell lines (Fig. 4E, F). Also in these cells, LAMP1 is unaffected. Finally, we tested whether LAMP2 upregulation is a common response to inhibition of nucleotide synthesis or is a specific response to purine synthesis inhibition. We therefore treated Hep3B cells with A771726, an inhibitor of dihydroorotate dehydrogenase and thus of pyrimidine synthesis, for 48 h and analyzed LAMP1 and LAMP2 protein expression. Unlike purine synthesis inhibitors, A771726 fails to increase LAMP2 (Fig. 4G), indicating that the response is specific to inhibitors of purine synthesis.

**Fig. 5:**
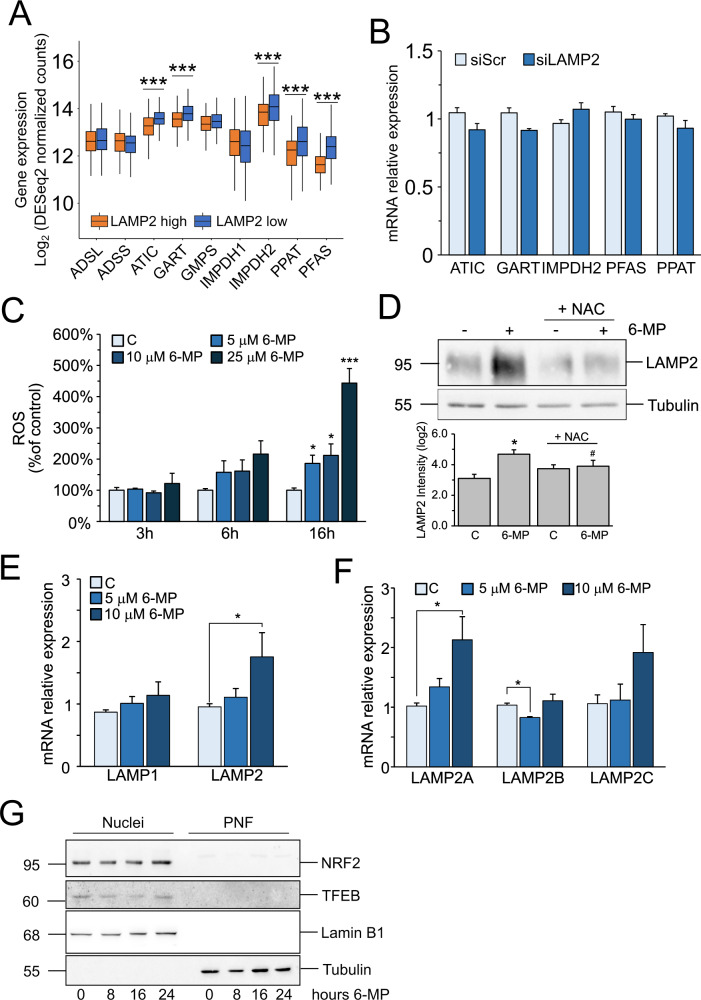
Inhibition of purine synthesis increases LAMP2 expression in a transcriptional- and Ros-dependent manner. **A** Expression of genes involved in purine synthesis in LAMP2-low and LAMP2-high cells. *p ≤ 0.001. **B** Real-time qPCR analysis of mRNA expression of genes involved in purine synthesis in siScr and siLAMP2 Hep3B cells. Data are expressed as the mean ± SEM of three independent experiments. **C** Fluorimetric analysis of intracellular ROS content using DCF-DA in Hep3B cells treated for the indicated times with the indicated drug concentrations. Data are expressed as the mean ± SEM of three independent experiments. *p ≤ 0.05 vs C; **p ≤ 0.01 vs C. **D** (Upper) Western blot analysis of LAMP2 in Hep3B cells pre-treated for 3 h with 5mM N-acetyl-L-cysteine (NAC) and then treated for 48 h with 10 μM 6-MP. (lower) Densitometric analysis of LAMP2 band intensities. Data are expressed as the mean ± SEM of log2(band intensity) of three independent experiments. *p ≤ 0.05 vs C; #*p* ≤ 0.05 vs 6-MP. Real-time qPCR analysis of mRNA expression of LAMP1 and LAMP2 (**E**) and LAMP2A, LAMP2B, and LAMP2C (**F**) in Hep3B cells treated for 48 h with the indicated drug concentrations. ACTB was used as the housekeeping gene. Data are expressed as the mean ± SEM of three independent experiments. *p ≤ 0.05 vs C; **p ≤ 0.01 vs C. **G** Nuclear localization of NRF2 and TFEB in Hep3B treated with 10 μM 6-MP for the indicated times. Lamin B1 and Tubulin were used as controls of nuclear and post-nuclear (PNF) fractions, respectively. 6-MP 6-mercaptopurine.

Finally, we tested whether the relationship between purine synthesis and LAMP2 was bidirectional and analyzed the expression of genes involved in purine synthesis in LAMP2-high and LAMP2-low cells used for the in silico screening (Fig. 1A). The results (Fig. 5A) showed that some genes are significantly upregulated in cells expressing low LAMP2 levels. However, qPCR experiments in siLAMP2 cells did not confirm these data and no significant difference in gene expression between siScr and siLAMP2 cells could be evidenced (Fig. 5B).

### Increase of LAMP2 expression is transcriptional- and ROS-dependent

LAMP2 expression has been shown to be increased by stress conditions, such as nutrient deprivation and oxidative stress [19]. Fluorimetric analysis of ROS levels using the fluorescence probe DCF-DA in Hep3B cells treated with 6-MP shows a significant time- and dose-dependent accumulation of ROS (Fig. 5C). Blocking of ROS with the antioxidant molecule N-acetyl-L-cysteine (NAC) reduced 6-MP-induced accumulation of LAMP2 (Fig. 5D), indicating that LAMP2 accumulation is ROS-dependent. Finally, we analyzed LAMP2 mRNA levels upon treatment with 6-MP by Real-Time qPCR, to verify whether the upregulation of LAMP2 occurs at the transcriptional level. Figure 5E shows that 6-MP upregulates LAMP2 but not LAMP1 mRNA levels, confirming the results obtained with WB (Fig. 4). Further analysis of the three LAMP2 isoforms (LAMP2A, LAMP2B and LAMP2C) shows that only LAMP2A mRNA is significantly upregulated (Fig. 5F). Since we observed an increase in LAMP2 mRNA, we investigated whether two known transcription factors upstream of LAMP2, the Nuclear factor erythroid 2-related factor 2 (NRF2) and the Transcription factor EB/Transcription factor E3 (TFEB/TFE3) could be the responsible. We performed nuclei isolation in Hep3B cells treated with 10 μM 6-MP for different time and analyzed the expression of NRF2 and TFEB in the nuclear fraction. Both are predominantly localized in the nucleus of the cells but neither of them is influenced by 6-MP treatment (Fig. 5G), indicating that they are likely not involved in the regulation of LAMP2 in our experimental conditions.

## Discussion

About 100 years ago Otto Warburg made the groundbreaking discovery that cancer cells preferentially use anaerobic glycolysis instead of the more efficient aerobic glycolysis to sustain their growth, also in the presence of normal oxygen concentration [20]. This phenomenon, known as the Warburg effect, revealed that cancer cells are not merely cells that proliferate faster than normal cells, but they are substantially different in several aspects, with metabolism being one of them. About two decades later Sidney Farber, the father of modern chemotherapy, successfully employed antimetabolites interfering with DNA synthesis to treat children with acute lymphoblastic leukemia [21], providing the first demonstration that metabolic reprogramming represents a vulnerability of cancer cells and an appealing therapeutic target. Since then, drugs targeting cancer cells metabolism have been used or tested for the treatment of several cancer types [22]. Intrinsic resistance and adaptation mechanisms to drug exposure are common events that limit the efficacy of anticancer strategies, eventually leading to tumor relapse and chemoresistance [23, 24]. It is therefore crucial to understand the determinants of tumor sensitivity to specific drugs and the mechanisms responsible for chemoresistance.

In this context, lysosomes have a key role in maintaining cellular homeostasis and adaptation to stress conditions [25], and lysosomes of cancer cells undergo profound changes in size, composition and localization to support cancer development, progressions and chemoresistance [3]. Expression of LAMP1 and LAMP2, the most abundant lysosomal membrane proteins, has been shown to be either upregulated or downregulated in cancer and associated with chemoresistance [6, 7]. We thus wondered whether it is possible to use LAMPs expression as a predictor marker of cell sensitivity to specific drug types. By combining gene expression data with gene dependency and drug efficacy screenings we identified inhibitors of de novo purine synthesis as a drug class whose toxicity inversely correlated with LAMP2 expression. To validate the in silico data, we selected as the main cellular model the HCC cell line Hep3B. We selected HCC because it is one of the most common and deadliest cancers worldwide, has limited therapeutic options, and in which purine synthesis was shown to be upregulated and considered a potential therapeutic target [12, 17]. It is therefore important to discover the determinants of drug efficacy and potential resistance mechanisms in this type of cancer. In vitro experiments showed that manipulating LAMP2 expression influences the cytotoxic effect of purine synthesis inhibition in Hep3B cells, confirming our prediction and the validity of our approach. The effect of LAMP2 is independent of autophagy, of which lysosomes are the terminal station. Indeed, the inhibition of autophagy by ATG7 knockdown fails to reproduce the effect of LAMP2 knockdown on cell viability, and the levels of LC3-II upon chloroquine treatment are comparable in 6-MP-treated and control cells, an indication that autophagy is not induced. On the contrary, 6-MP treatment strongly reduces LC3 levels in the absence of chloroquine and increases intracellular acidification, suggesting that lysosomal activity is increased.

Mechanistically, we demonstrated that inhibitors of purine synthesis upregulate LAMP2 mRNA and protein levels. LAMP2 plays several important functions in the lysosomes, including the preservation of lysosomal membrane stability [19], the regulation of the late stages of macroautophagy and of the lysosomal import of specific proteins via chaperone-mediated autophagy [18, 26]. The isoform C of LAMP2 (LAMP2C) has also been shown to directly bind both RNA and DNA and to act as a lysosomal receptor for RNautophagy/DNautophagy [27]. However, our data shows that only the isoform A of LAMP2 (LAMP2A) is significantly upregulated in our experimental conditions. LAMP2C shows an increasing trend that does not reach significant levels. It is also worth mentioning that LAMP2C is expressed at very low, almost undetectable levels in liver-derived cells, as we observed in the amplification plots of our Real-Time qPCR experiments (data not shown) in Hep3B cells and it was shown in a previously published paper by another group [28].

A pending question is how the inhibition of purine synthesis triggers LAMP2 expression and lysosomal activity. Lysosomes are a nutrient-sensing center and their functions are regulated by nutrient availability [29], but because LAMP2 upregulation is a specific response to inhibitors of purine synthesis but not pyrimidine synthesis, it is unlikely that LAMP2 expression is triggered by the reduced availability of substrates for DNA or RNA synthesis. The purine inhibitors that we used have different mechanisms of action, but they all upregulated LAMP2. Indeed, 6-TG and 6-MP are nucleotide analogs that block purine synthesis by pseudofeedback inhibition of glutamine-5-phosphoribosylpyrophosphate amidotransferase, the first enzyme of purine synthesis [30], while MPA is a specific inhibitor of inosine-5′-monophosphate dehydrogenase, which controls the synthesis of guanine nucleotides. It is thus possible that LAMP2 upregulation is triggered by an active signaling mediated by alterations of purine synthesis intermediates, such as xanthosine monophosphate and inosine-5′-monophosphate, or GTP-dependent proteins. Additional analyses are also required to clarify the upstream factor(s) responsible for LAMP2 upregulation. Two known LAMP2 upstream regulators, NRF2 and TFEB, are not influenced by the inhibition of purine synthesis. Other transcription factors or epigenetic regulators like the Bromodomain Containing 4 (BRD4) could be involved [31–34]. It is important to note that LAMP2 is often modulated in a concerted manner with LAMP1, while in our case LAMP1 remains unchanged, suggesting the existence of additional, LAMP2-specific upstream regulators or a more intricated regulatory network leading to the specific regulation of LAMP2. Overall, our results reveal that LAMP2 upregulation represents an adaptive mechanism to purine synthesis inhibition and the factors upstream of LAMP2, once identified, will represent a target for combination therapy to prevent the appearance of chemoresistance. Additionally, high LAMP2 expression may also represent an intrinsic mechanism of resistance to inhibitors of purine synthesis in those cancer types in which LAMP2 is upregulated. LAMP2 expression in tumors could eventually be taken into consideration during the selection of the most appropriate anticancer therapy.

## Materials and methods

### Cell culture

Hep3B and HepG2 cells were purchased from the Leibniz-Institut DSMZ (ACC-93, ACC-180). HuH7 cells were purchased from Cytion (300156). Hep3B and HuH7 were grown in DMEM Low Glucose (Euroclone, ECM0749L), and HepG2 were grown in RPM1640 (Euroclone, ECB9006L), all supplemented with 10% FBS (Euroclone, ECS0180L), 2 mM L-Glutamine, and 1% penicillin-streptomycin. Cells have been authenticated by STR PCR by the supplier and routinely tested for Mycoplasma contamination using Myco Alert (Lonza, LT07-318).

### Treatments

6-Mercaptopurine (6-MP; Merck Life Science, 852678), 6-Thioguanine (6-TG; Merck Life Science, A4882), and Mycophenolic acid (MPA; Santa Cruz Biotechnologies, sc-200110) were dissolved in 0.1 M NaOH and used at the final concentrations indicated in the text. A771726 (Santa Cruz Biotechnologies, sc-207235) was dissolved in DMSO and used at the final concentrations indicated in the text. Chloroquine (CQ; Merck Life Science, C6628) was dissolved in DMSO and used at the final concentration of 50 μM. N-acetil-L-cysteine (NAC; Merck Life Science, A9165) was dissolved in water, pH adjusted to 7.4 and used at the final concentration of 5 mM.

### Transfection

The following siRNAs (all from Merck Life Science) were used: LAMP2 (5′-ATTTGTAGTTTCATAGCGTAC-3′); ATG7 (5′-ATGGAGAGCTCCTCAGCAGGC-3′); ADSL (5′ -UAUUCAAUAUAGUAUCUGCGG-3′); siRNA Universal Negative Control (SIC001) was used as the control siRNA; all siRNAs were used at the final concentration of 25 nM. pcDNA3-LAMP2A plasmid was a gift from Prof. Renate Kain, Medizinische Universität Wien. All siRNAs and plasmids were transfected using Lipofectamine 3000 (Life Technologies), following the manufacturer’s instructions.

### Immunoblotting

Cells were lysed in RIPA buffer (150 mM NaCl, 25 mM Tris-HCl pH 7.4, 1 mM EDTA, 1 mM Na_3_VO_4_, 10 mM NaF, and protease inhibitor cocktail) and centrifuged at 14,000 × *g* for 15 min at 4 °C. Supernatants were collected, samples electrophoresed by SDS-PAGE and blotted onto a nitrocellulose membrane (GE Healthcare 10402495). Primary antibodies used were as follows (dilution 1:1000 unless otherwise stated): anti-PARP1 (#9542), anti-β-Actin (#4870S), anti-LC3B (#2775), and anti-ATG7 (#88577), anti-LAMIN B1 (#13435S), and anti-NRF2 (#12721S) were from Cell Signaling Technology; anti-LAMP1 (sc-20011), anti-LAMP2 (sc-18822), anti-TFEB (1:250, sc-166736), and anti-α-Tubulin (sc-5286) were from Santa Cruz Biotechnology. Immunoblots were acquired using a Fluorchem imaging system (Alpha Innotech) and quantified using the AlphaEaseFC software (Alpha Innotech).

### Real-time qPCR

Cells were lysed with TRItidy G (PanReac AppliChem) and RNA was extracted according to the manufacturer’s instructions. Total RNA was resuspended in RNase-free water and 1 µg of total RNA was used to generate cDNA using the iScript™ cDNA Synthesis Kit (Bio-Rad). Real-time PCR was performed using the PowerUp SYBR Green Master Mix (Thermo Fisher Scientific) on a QuantStudio 3 Real-Time PCR system (Thermo Fisher Scientific). The following primer pairs were used: LAMP1 (Forward: 5′-CGTGTCACGAAGGCGTTTTCAG-3′; Reverse: 5′-CTGTTCTCGTCCAGCAGACACT-3′); LAMP2 (Forward: 5′-GGCAATGATACTTGTCTGCTGGC-3′; Reverse: 5′-GTAGAGCAGTGTGAGAACGGCA-3′); LAMP2A (Forward: 5′- ACTGTTTCAGTGTCTGGAGCAT-3′; Reverse: 5′- GCACTGCAGTCTTGAGCTGT-3′); LAMP2B (Forward: 5′- GGGTTCAGCCTTTCAATGTGAC-3′; Reverse: 5′- GCCTGAAAGACCAGCACCAA-3); LAMP2C (Forward: 5′- GAAGGAAGTGAACATCAGCATG-3′; Reverse: 5′- CTCGAGTTACACAGACTGATA-3), PPAT (Forward: 5′-GCGATTGAAGCACCTGTGGATG-3′, Reverse: 5′-CGGTTTTTACACAGCACCTCCAC-3′); GART (Forward: 5′-CACCCGGTGTCGGTTTCA-3′; Reverse: 5′-TTCCAGGCCAGCGTATGTTC-3′); PFAS (Forward: 5′- TGAGGCTATGGTGGCAGTGATG-3′; Reverse: 5′-GCATAGGCTGAGATGACCAGTG-3′); ATIC (Forward: 5′-CCGAGAGTAAGGACACCTCCTT-3′; Reverse: 5′-GGCATCTGAGATACGCCTTTGC-3′); IMPDH2 (Forward: 5′-AGTGGCTCCATCTGCATTACGC-3′, Reverse: 5′-GGATTCCTCCATCAGCAATGACC-3′); ACTB (Forward: 5′- CCCCTGGCGGCCTAAGGACT-3′; Reverse: 5′-ACATGCCGGAGCCGTTGTCG-3′). All reactions were run as triplicates. Data were analyzed by the Design and Analysis Application (Thermo Fisher Scientific) using the second derivative maximum method. The fold changes in mRNA levels were relative to the control after normalization with β-Actin (ACTB).

### Immunofluorescence

Cells were seeded on coverslips and treated 24 h later. Cells were then fixed for 15 min with 10% formalin solution (HT501128, Merck Life Science), solubilized for 20 min with PBS/digitonin (100 μg/mL) and incubated for 30 min with a blocking solution (PBS-BSA 1%). Cells were incubated with LAMP2 antibody (sc-18822, Santa Cruz Biotechnologies; 1:100 dilution) for 1 h at RT and with a specific Alexa Fluor™ 568 secondary antibody (Thermofisher; 1:1000) for 1 h at RT in the dark. Nuclei were stained for 10 min with Hoechst 33342 (52401, Enzo Life Sciences; 1:10000). Images were acquired using an Axio Observer microscope (Zeiss) equipped with a Plan-Apochromat 63x/1.46 oil objective lens and connected to a Zeiss AxioCam. Quantification of LAMP2 fluorescence intensity was performed using the Zen Blue software.

### Flow cytometry

To analyze intracellular acidification, which mostly depends on lysosomes, 1 μg/mL Acridine Orange (318337, Merck Life Science) was added to cells 10 min before the end of the experimental time. Cells were then washed with PBS, mechanically detached, and analyzed with a BD FACSCalibur™ Flow Cytometer.

### Live cell counting

At the end of the experimental time cells were collected and live cells counted after staining with 0.08% Trypan Blue (T8154; Merck Life Science).

### MTT assay

Five thousand cells were seeded in 96-well plates and treated 24 h later. Then, 200 μL/well of fresh medium supplemented with 20 μL of 5 mg/mL 3-(4,5-Dimethylthiazol-2-yl)-2,5-diphenyltetrazolium bromide (MTT) (88417, Merck Life Science) were added. After 2 h of incubation at 37 °C medium was removed and 100 μL of DMSO were added to each well. Absorbance was measured at 570 nm.

### ROS analysis

To analyze intracellular ROS, 1 × 10^5^ cells were plated in a 24-well plate. The next day, cells were treated with 6-MP for up to 16 h. At the end of the experimental time, cells were incubated for 30 min with 20 μM 2’,7’-dichlorofluorescein diacetate (DCF-DA, D399, Thermo Fisher Scientific), washed three times with PBS and lysed with 50 μL of RIPA Buffer. After centrifugation at 14,000 × *g* for 15 min at 4 °C, supernatants were transferred to flat bottom black 96-well plates and DCF fluorescence intensity measured using a Tecan Sunrise™ microplate reader at an excitation/emission wavelength of 485/520 nm. Protein concentration was used to normalize the values.

### Nuclei isolation

Cells were lysed in a nucleus extraction buffer (NB; 0.25 M sucrose, 10 mM Tris-HCl pH 8, 10 mM MgCl2, 1 mM EDTA, 1% Triton X-100 and 0.5 mM DTT) containing protease/phosphatase inhibitors. After centrifugation at 600 × *g* for 10 min at 4 °C, supernatants were transferred to new tubes and pellets containing nuclei were resuspended with NB and centrifuged at 600 × *g* for 10 min at 4 °C. Pellets were then lysed in RIPA buffer and processed for SDS-PAGE.

### Bioinformatic analyses

To identify potential gene candidates whose essentiality is dependent on LAMP2 expression, cell lines included in the Combined Achilles and Sanger Score Data (21Q1) have been ranked according to LAMP2 expression, using expression data obtained from the Cancer Cell Line Encyclopedia (CCLE) and expressed as DESeq2 normalized counts. Cell lines were divided into two groups: LAMP2 high, containing the cell lines of the top 25% of the ranking, and LAMP2 low, containing the cell lines of the bottom 25% of the ranking. The difference (LAMP2 low—LAMP2 high) of the mean gene essentiality score of cell lines of each group has been calculated. A difference <0 indicates a higher sensitivity of LAMP2 low cells to gene knockout, while a difference >0 indicates a higher sensitivity of LAMP2 high cells. The same approach has been used to identify drug sensitivity, using data from the PRISM Repurposing Primary Screen. All datasets are freely available on the DepMap Portal (https://depmap.org/portal/).

The identification of enriched Gene Ontology (GO) terms in the Biological Processes (BP) category has been performed using the Database for Annotation, Visualization, and Integrated Discovery (DAVID) tool (https://davidbioinformatics.nih.gov/) [35, 36]. Genes with a difference of mean essentiality ≤ - 0.1 and a q-value ≤ 0.05 have been used as the input.

### Statistical analysis

For experiments with cell lines, values are expressed as the mean ± SEM of 3 independent experiments, unless otherwise stated; statistical significance of pairwise differences was calculated using the two-tailed Student’s *t* test. A *p* value ≤ 0.05 was considered statistically significant. For bioinformatics analyses, raw counts of expression data of the Cancer Cell Line Encyclopedia (CCLE) have been normalized using DESeq2. Correction of the *p* values was performed using the Benjamini-Hochberg procedure (*q* value). All analyses have been performed using packages of the R programming language.

## Supplementary information

UNCROPPED BLOTS

## Data Availability

Original data are available upon reasonable request. The full-length uncropped blots are shown in the ‘Supplementary Material’.
