# Supplementary material for: The inhibition of de novo purine synthesis increases LAMP2 expression to preserve cell viability
Source: Cell Death Discov. 2025 Dec 11;12:42. doi: 10.1038/s41420-025-02884-0 (PMC12830960; doi:10.1038/s41420-025-02884-0)

Figure 2

Panel D

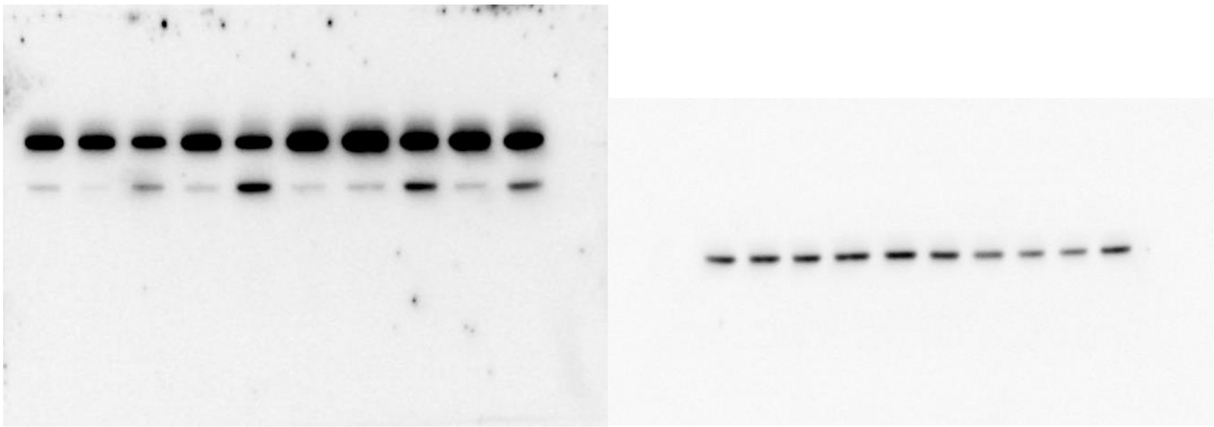

Panel F

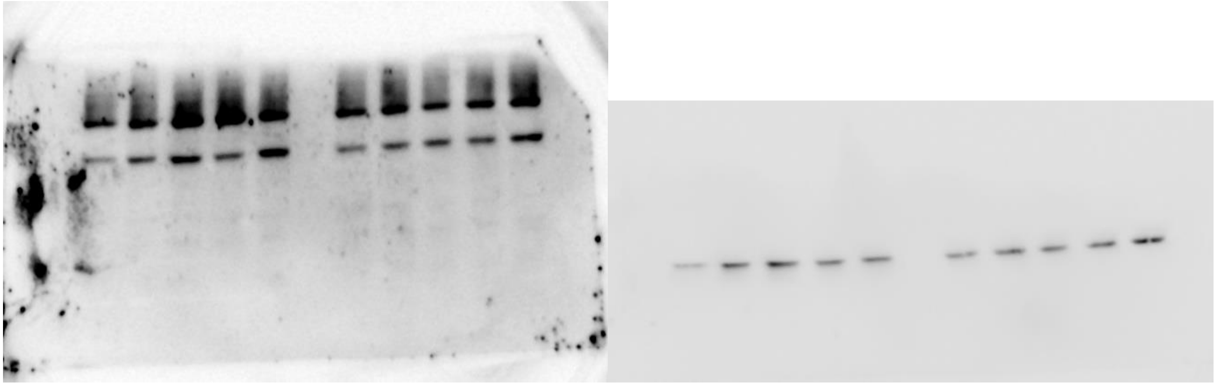

Figure 3

Panel B

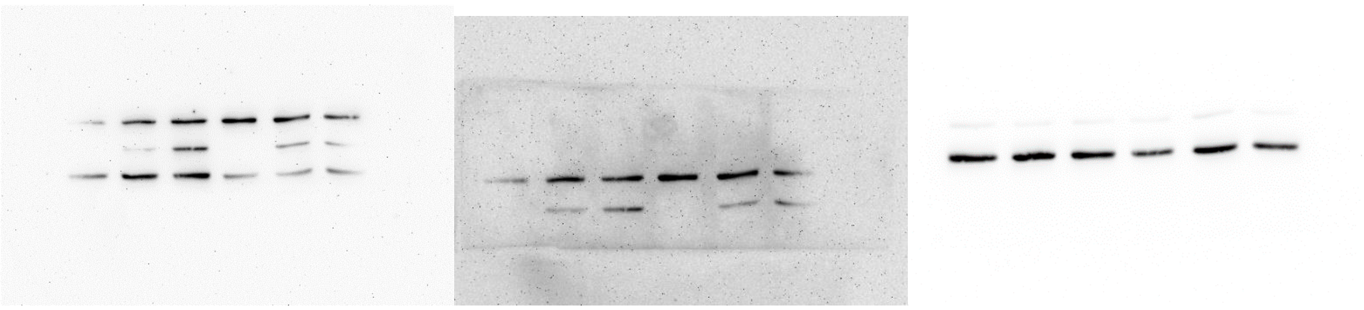

Panel E

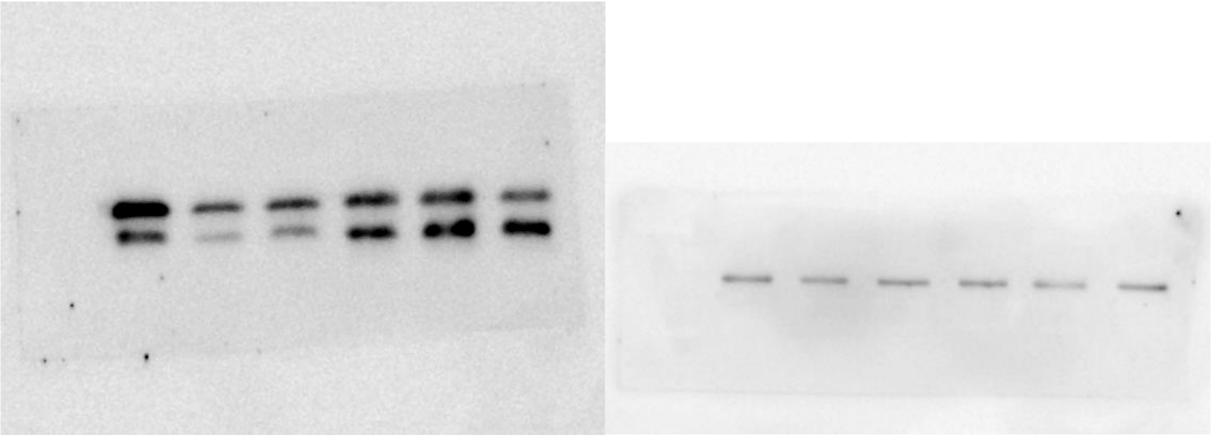

Figure 4

Panel A

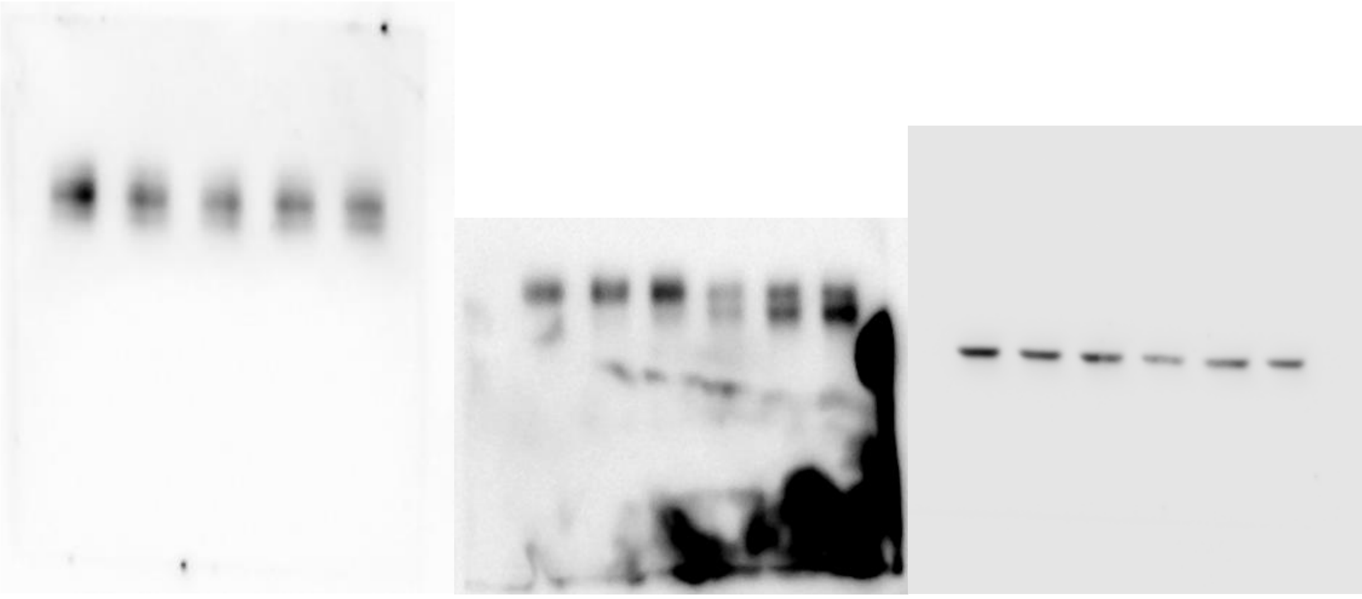

Panel B

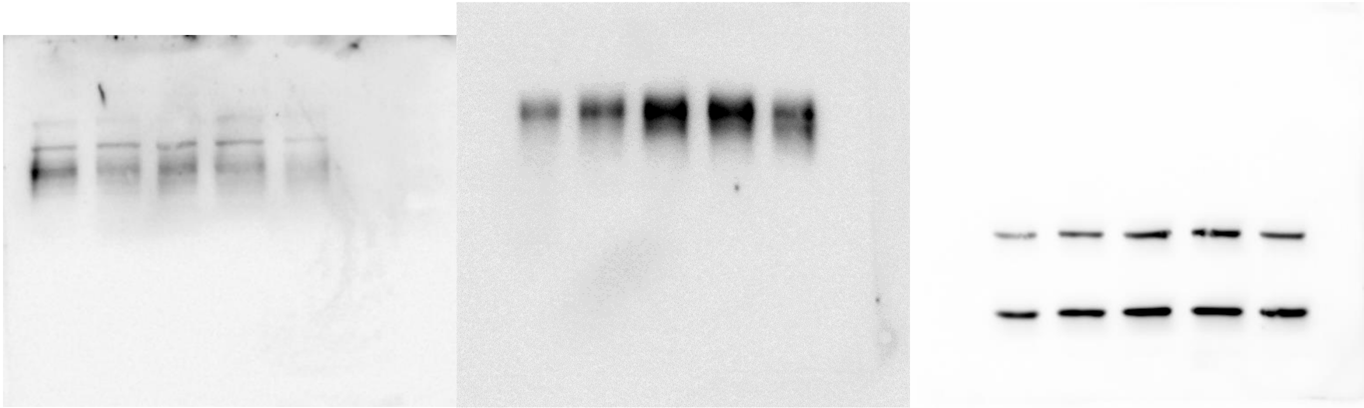

Panel C

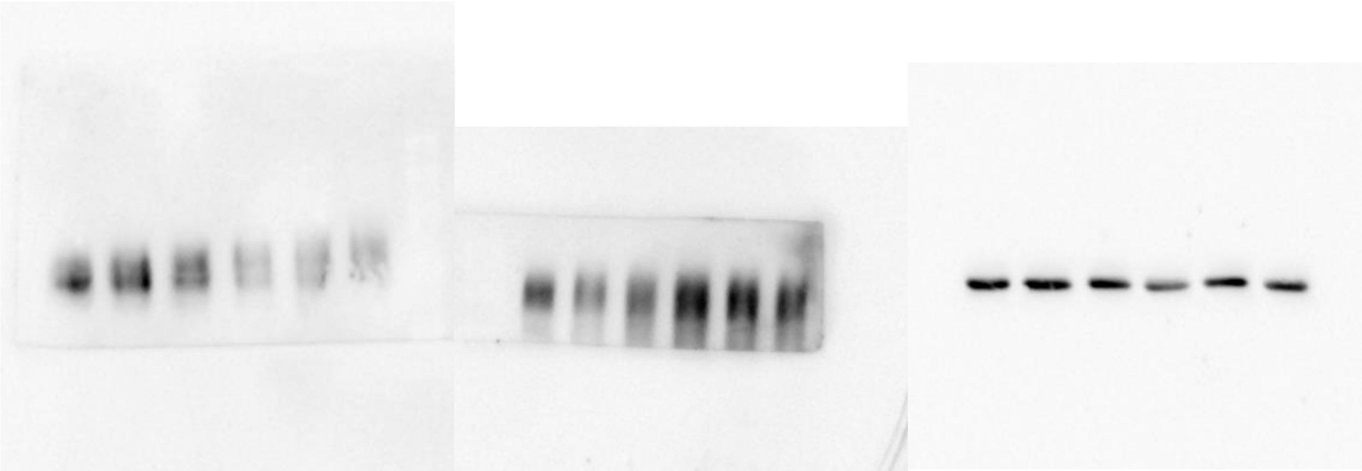

Panel E

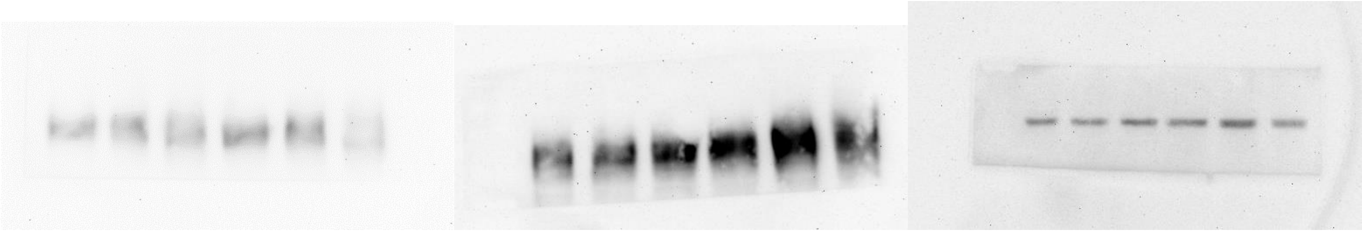

Panel F

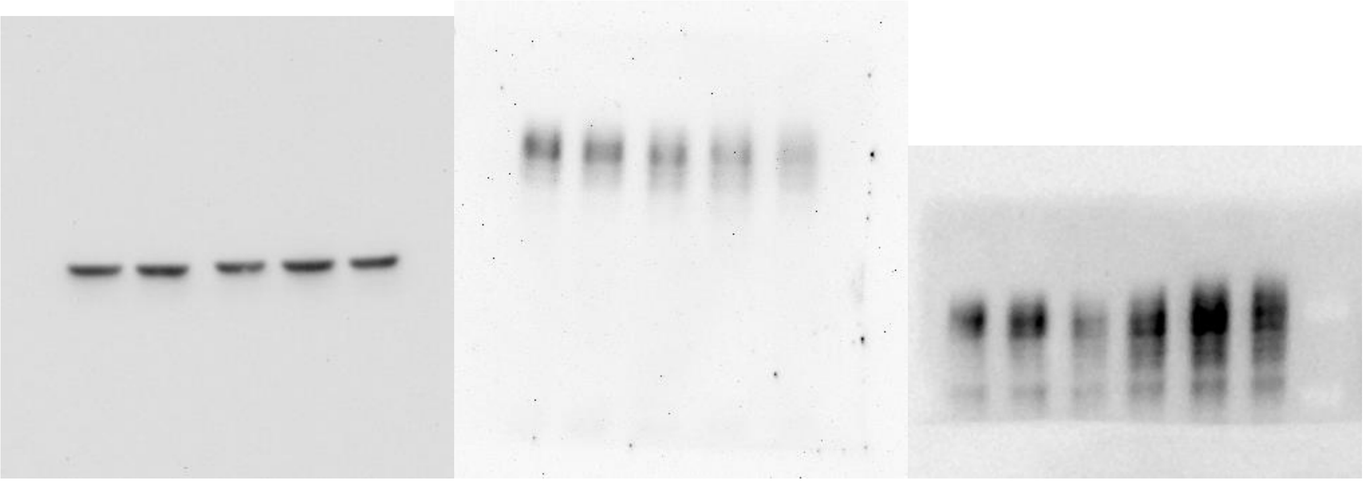

Panel G

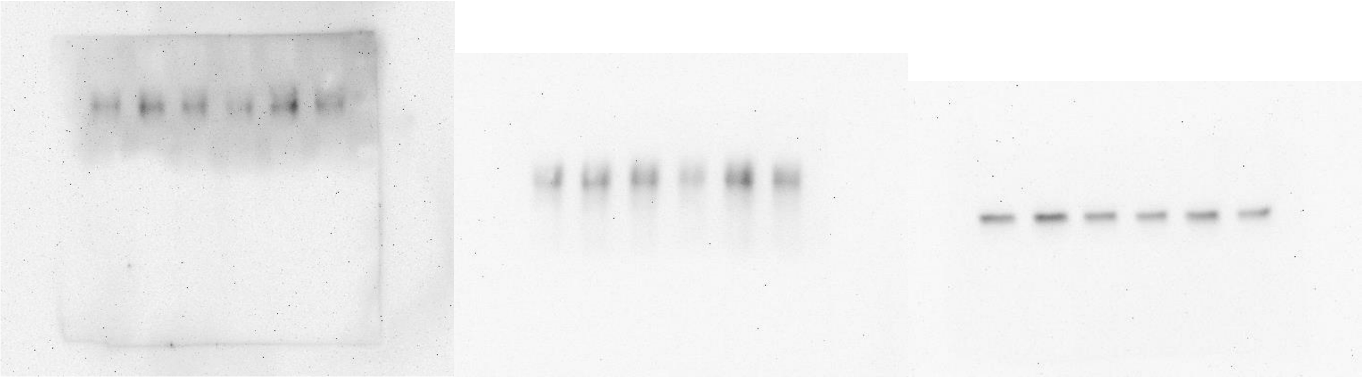

Figure 5

Panel D

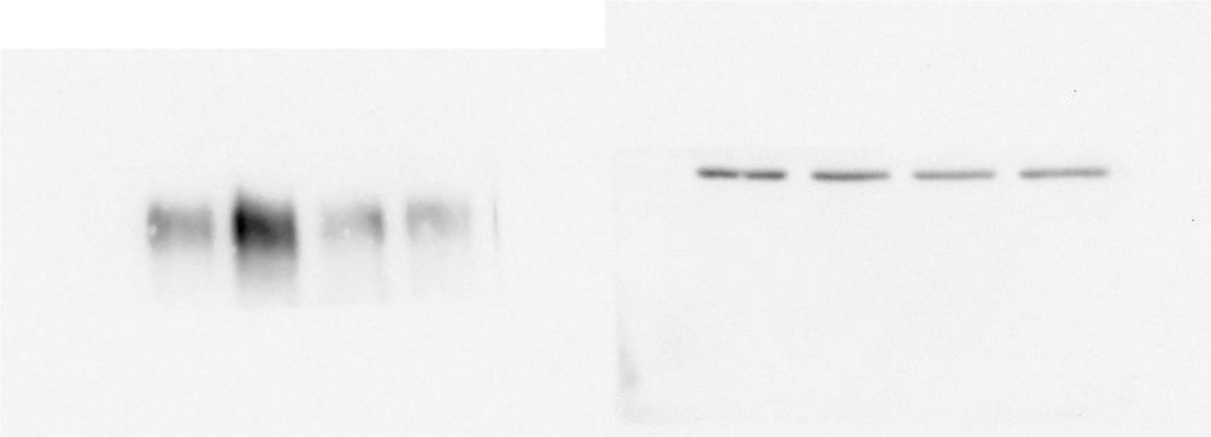

Panel G

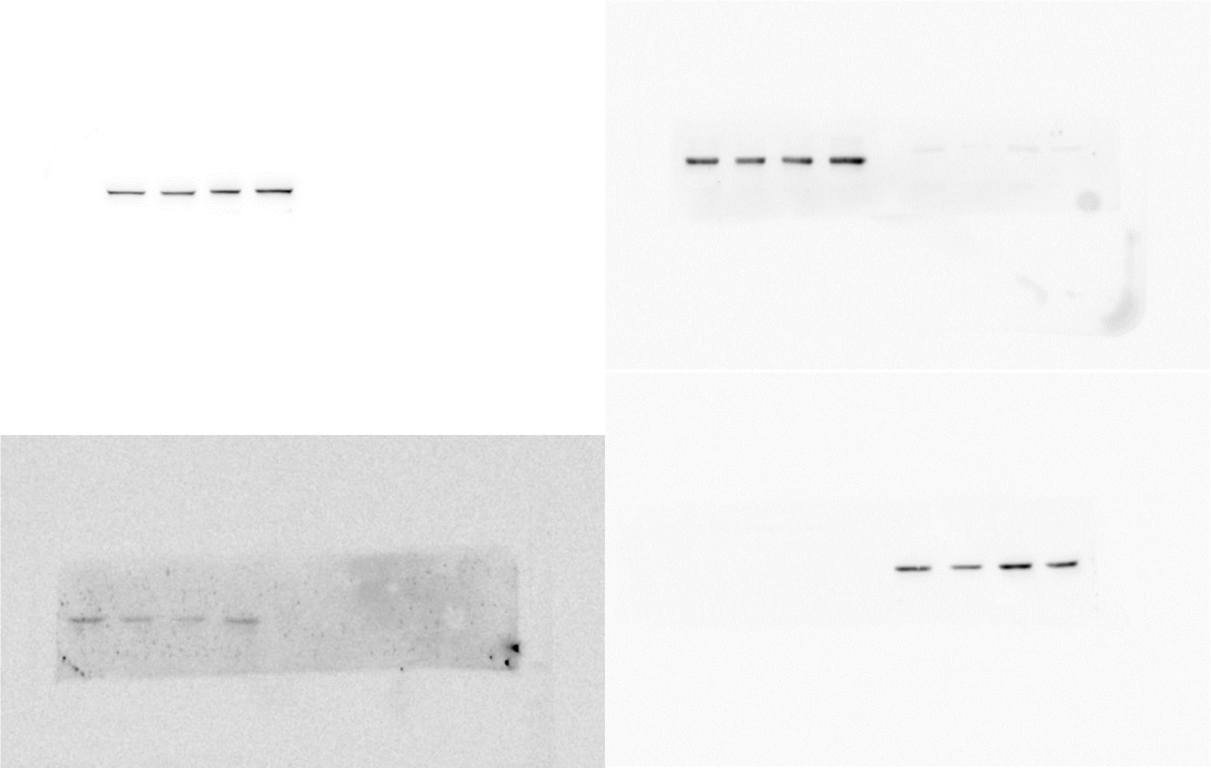

Supplement: Supplementary file 1 — UNCROPPED BLOTS [file 41420_2025_2884_MOESM1_ESM.pdf]
